# Protective effect of irisin against Alzheimer’s disease

**DOI:** 10.3389/fpsyt.2022.967683

**Published:** 2022-09-20

**Authors:** Kang Chen, Kun Wang, Tianhui Wang

**Affiliations:** ^1^Tianjin Key Lab of Exercise Physiology and Sports Medicine, Tianjin University of Sport, Tianjin, China; ^2^Institute of Environmental and Operational Medicine, Academy of Military Medicine Sciences, Academy of Military Sciences, Tianjin, China

**Keywords:** irisin, Alzheimer’s disease, BDNF, neuroprotection, neuroinflammation

## Abstract

Despite being one of the greatest global challenges for health and social care in the 21st century, Alzheimer’s disease (AD) lacks specific medicine. Irisin, an exercise-generated muscle factor, emerges as a potential hormone for AD prevention and treatment because of its role in promoting the browning of white adipose tissue, accelerating energy expenditure, regulating energy metabolism, and improving insulin resistance. The study reviews classic hallmarks of AD and irisin’s physiology before discussing the possible mechanism by which irisin protects against AD in terms of its effects related to molecular biology and cellular biology. Results reveal that irisin sharpens learning memory by inducing the production of brain-derived neurotrophic factor (BDNF), lowers the production of inflammatory factors, protects neurology through astrocytes, and ameliorates AD symptoms by improving insulin resistance. The review aims to facilitate future experimental studies and clinical applications of irisin in preventing and treating AD.

## Introduction

Alzheimer’s disease, as a neurodegenerative disease, impacts not only patients and their families but also social security (1, 2). World Health Organization revealed that over 55 million people (8.1% of women and 5.4% of men over the age of 65) worldwide suffered from dementia in 2021. AD is the first to blame when it comes to disability in the elderly, and its incidence grows exponentially with age. It also requires higher treatment costs compared with heart diseases and cancers, increasing the financial burden on the global economy and patients’ families (3). Dr. Alois Alzheimer first reported AD and its features of progressive memory loss and other cognitive impairments as early as 1907 (4), but no definitive therapy was discovered.

The neurodegenerative disease has complicated and diverse causes represented by genetic factors and aging (5), and largely unknown mechanisms (6). Multiple studies proved the effectiveness of exercise in reducing the risk factors for AD (7–9). A growing body of evidence shows the higher risk of AD caused by inadequate physical exercise, obesity, high blood pressure, and diabetes, and interventions in such factors prevent or delay 40% of dementia (10). Klodian et al. stated the close relationship between risk factors and incidence (11). Exercise helps reduce common risk factors among patients with AD and exhibits higher adherence than medication interference because of its fewer side effects (7, 8). The two main pharmacological treatments for AD, namely, cholinesterase inhibitors and *N*-methyl-*D*-aspartate amino receptor blockers in clinical trials, fail to improve symptoms significantly (12, 13), indicating the importance of alternative forms of treatment and prevention methods such as exercise. Alzheimer’s Disease International calls for 150-min of moderate aerobic exercise or 75-min of vigorous aerobic exercise each week among adults for normal heart function, blood circulation, weight management, and mental health. There is also evidence that exercise can be an effective intervention in early AD (7, 14, 15).

Recently, many studies have discovered that irisin produced by exercise can improve cognition through various pathways. Specifically, irisin promotes neurogenesis by BDNF, increases neuronal survival and synaptic plasticity (16), reduces the release of inflammatory factors IL-6 and IL-1β, decreases Aβ protein and tau protein formation, and undermines insulin resistance (15, 17). These factors indicate that irisin could potentially play a significant role in the treatment and prevention of AD. This review discusses irisin’s role in AD, highlighting irisin’s mechanism of action in relation to AD pathology and assessing the current evidence base for the use of irisin as a treatment for AD.

## Alzheimer’s disease and irisin

### Classic hallmarks of Alzheimer’s disease

#### The amyloid hypothesis

Aβ protein deposition serves as an essential part of classic hallmarks for AD, and Aβ refers to a polypeptide formed by amyloid precursor protein hydrolysis by β and γ secretases. According to animal experiments, rats, which were injected with Aβ and underwent the Morris water maze test, performed decreased spatial learning and memory of Aβ. Hardy and Higgins first proposed the protein cascade hypothesis in 1992, which regarded amyloid deposition as the primary risk factor for AD. Aβ deposition triggers the dysfunction and death of neurons in the brain, crippling normal brain function. In particular, the imbalance of Aβ protein metabolism directly stimulates the production of inflammatory factors in neurons, leading to neuroinflammation and oxidative stress. Meanwhile, Aβ protein activates microglia and astrocytes around senile plaques, releasing pro-inflammatory factors such as TNF-α or IL-1-β (18). Prolonged neuroinflammation leads to neurosynaptic dysfunction, thus accelerating neuronal degeneration, exacerbating brain damage, and causing lesions. The interaction between Aβ and glial cells is supposedly mixed. On the one hand, glial cells reduce Aβ protein deposition through phagocytic clearance and degradation to protect neurons and prevent amyloid plaque formation in the brain. On the other hand, Aβ protein activates glial cells and facilitates the sustained release of inflammatory cytokines, which promotes Aβ protein deposition and neuroinflammatory responses, incurs immune responses, and accelerates the AD pathological process. In addition, Aβ protein deposition decreases the level of proteins that specialize in the long-term potentiation effect in the hippocampus, which explains the progressively impaired learning memory capacity of patients with AD. Recent studies have also found that Aβ protein aggregation can affect the mitochondrial function of neuronal cells, thereby causing mitochondrial damage and leading to oxidative stress (19). Excessive oxidative stress not only hinders mitochondrial electron transfer but also damages mitochondrial DNA and related synapses. Notably, serious mitochondrial damage accelerates the apoptosis of associated neuronal cells and worsens the condition. In addition, Aβ protein deposition also decreases CBF (20), which is assumed to inhibit mitochondrial ATP production. Studies that supported the hypothesis proved the accumulation of soluble Aβ protein in the cerebral vasculature and the following decline in CBF and vasoconstriction, which compromises neuronal effort. Overall, by stimulating nerve cells, Aβ releases inflammatory factors that promotes neuronal dysfunction and death and affects the normal function of mitochondria and CBF, thus denting the normal function of the brain.

#### The tau hypothesis

One of the pathological features of AD is the presence of neurofibrillary tangles (NFTs) in neuronal cells, whose main component is the abnormally phosphorylated tau protein. It refers to a phosphorylated glycoprotein closely related to microtubule structure and is essential for the synthesis and maintenance of the neuronal skeleton and intracellular transmission of substances and information between neurons. Over-phosphorylation of tau protein, however, disrupts neuronal microtubule structure, reduces the binding to microtubule proteins, and hinders microtubule formation and microtubule stability (21, 22), which results in the formation of NFTs and AD development. NFTs impair axonal transport function, causing synaptic loss, cytoskeletal and mitochondrial dysfunction, and memory loss. Therefore, hyperphosphorylated soluble tau protein may be responsible for AD neurotoxicity (23). The quantity and distribution of NFTs in the brain of patients with AD are higher than that of normal elderly, and NFTs climb with AD development and positively correlate with the degree of clinical dementia, which explains the general application of NFTs accumulation in the patient’s brain as one of the critical indicators of AD development degree in clinical tests (24). Some new therapeutic approaches, such as combined drugs targeting tau protein and Aβ, are vital for tau-related pathological processes in AD (25). In summary, tau protein phosphorylation in the brain of patients with AD leads to NETs that deepen the degree of AD pathology by affecting the transport function of neurons and the normal function of mitochondria.

#### Neuroinflammation of Alzheimer’s disease

Clinical studies have shown that neuroinflammation is a key pathological mechanism in AD (26). Neuroinflammation and activation of microglia and astrocytes are critical to the pathogenesis of AD (27). Shen et al. explored the difference in cerebrospinal fluid (CSF) or peripheral blood inflammatory markers between patients with AD and MCI and proved the higher level of interleukin-6 (IL-6), interleukin-10 (IL-10), and soluble tumor necrosis factor receptor 1 (sTNFR1) in the brains of patients with AD compared with patients with MCI (28). In addition, peripheral inflammation is one of the contributors to neuroinflammation in the brain, and pro-inflammatory cytokines have been shown to cross the blood-brain barrier, which in turn triggers a central inflammatory response. Damage to the blood-brain barrier also exacerbates central inflammation, allowing the entry of peripheral immune cells into the brain and promoting inflammation there. Besides, obesity is a critical factor in peripheral systemic inflammation, as studies have proved that excess saturated fatty acids in obese patients can cross the blood-brain barrier to directly affect neuroglia activation in the brain, and type 2 diabetes caused by obesity accelerates memory dysfunction and neuroinflammation in mouse models of AD (29). Neuroinflammation and β-amyloid deposition are causal, and even to the extent that neuroinflammation determines the pathophysiological process of β-amyloid. β-amyloid deposition in the brain results in continuous activation of glial cells, followed by neuroinflammatory factor release and inflammatory response. Dysfunctional energy metabolism in the brain also induces neuroinflammation. Mitochondrial dysfunction not only generates high levels of reactive oxygen species followed by microglia activation but also discourages the neuroprotective function of astrocytes, the latter of which comes down to astrocytes’ reliance on functional mitochondria for energy (30, 31). The neuroinflammation accordingly plays a pivotal role in AD. Several epidemiological studies have confirmed the role of non-steroidal anti-inflammatory drugs (NSAIDs) in preventing AD. Specifically, patients who used NSAIDs for more than 2 years had a 60% lower risk of AD, and those for less than 2 years had a 35% lower risk (32). Another study discovered an approximately 55% risk reduction in patients who had used NSAIDs for more than 2 years (33). In summary, neuroinflammation can increase the risk factors for AD, which should be tackled with inflammation reduction in the future.

#### Insulin resistance in the brain

Insulin refers to a peptide secreted by the pancreas whose regular expression benefits cognition. Insulin resistance, however, is prevalent in the brain of patients with AD (34). Insulin regulates glucose metabolism in peripheral tissues, enhances synaptic viability and dendritic spine formation, and increases neurotransmitter turnover through brain bioenergetics. Besides, its downstream signaling molecules are mainly distributed in the cerebral cortex and hippocampus (35). Insulin in the brain controls glucose and lipid metabolism, affects neurodevelopment, and sways learning and memory processes (34), while insulin/IR signaling in the central nervous system improves cognitive function, including learning and memory. The impairment of hippocampal insulin signaling in the brain of patients with AD undermines memory and other executive functions because of the concomitant decline in insulin signaling and insulin resistance. Decreasing insulin/IGF-1 levels and insulin/IGF-1 receptor deficiency has been found in models of aging and AD (36). Peripheral insulin resistance decreases insulin signaling in the central nervous system, that is, central insulin resistance, which disrupts brain metabolism and increases Aβ toxicity. Furthermore, insulin resistance prompts tau hyperphosphorylation, oxidative stress, and neuroinflammation, which ultimately results in neurodegeneration. Autopsy analysis of AD brains found increased dystrophic synapses and Aβ plaques but decreased levels of IGF-1 neurons. Some studies indicated the decline in energy metabolism that patients with AD experienced before cognitive dysfunction. Furthermore, the degeneration and loss of neurons in the brain of patients with AD aggravate abnormal glucose metabolism (36). Therefore, modulation of energy metabolism may be an important target for AD treatment.

## Effects of irisin against Alzheimer’s disease

### Irisin’s physiology

Irisin is a muscle factor discovered in 2012 by Bostrom et al. They found that exercise upregulated the gene expression of fibronectin type III domain-containing protein 5 (FNDC5) in skeletal muscle. FNDC5 was expressed and modified into a new small molecule, irisin, and released into the blood. Jedrychowski et al. identified and measured human irisin in plasma using mass spectrometry, determining its level in circulating blood to be 3.6–4.3°ng/ml (37). Ruan confirmed the higher level of irisin in the elderly and men than in the young and women, respectively, among an average population. Irisin in the cerebrospinal fluid was also positively correlated with BDNF and Aβ (38, 39). Irisin, a glycosylated protein hormone that consists of 112 amino acid residues, is highly conserved structurally and 100% homologous in mice and humans (40). Huh et al. scanned the distribution of FNDC5 in humans using tissue qPCR analysis microarrays and revealed the high expression of FNDC5 in skeletal muscle (41). Xie et al. detected FNDC5 gene expression in 47 human tissues, such as cardiac and smooth muscle, endothelium, adipose tissue, the liver, kidney, and pancreas, among which, after exercise, cardiac FDNC5 was highly expressed, and more irisin was produced in cardiac muscle than in skeletal muscle (42). Meanwhile, irisin is vital for the human body, and exercise-produced irisin has been proven to intervene in AD in several ways (7). Specifically, irisin can accelerate metabolism (represented by irisin browning of white fat and improving glucose homeostasis), cross the blood-brain barrier, and initiate neuroprotective genetic programs in the hippocampus, which incurs higher expression of BDNF and promotes synaptogenesis and neuronal cell survival (43). In addition, the multiple neuroprotective effects of irisin against injury in AD models have been proven. Recent studies confirmed the association between irisin in the risk factor of AD and the lower irisin level in the hippocampus and cerebrospinal fluid (CSF) of patients with AD (39). In contrast, the relation between higher irisin levels in the CSF and better cognitive function, and less amyloid-β pathology is observed in both patients with AD and non-AD (38). The relationship between blood levels of irisin and cognitive function, however, remains unknown given the identity of irisin as a hormone. Tsai et al. explored the circulating level of irisin and neurocognitive performance of thirty-two individuals with a family history of AD (ADFH) and obesity (ADFH-obesity group) and 32 controls (ADFH-non-obesity group) during a visuospatial working memory task and discovered the direct proportion between serum irisin levels and cognitive function in cognitively normal individuals, which indicates the protective role of irisin in the brain (44). In contrast, subsequent studies obtained the opposite results that serum irisin levels did not differ between cognitively normal and AD individuals (45). The above suggests the different effects of irisin on brain cells in the AD brain. The varied roles of irisin in different degrees of AD validate its effective intervention in AD.

### Irisin enhances learning memory by promoting the expression of brain derived neurotrophic factor

Exercise-induced irisin can cross the blood-brain barrier with the help of peripheral transport before entering the central nervous system and inducing BDNF expression (46). The receptor of irisin in the neonatal brain is reported to be an integrin αV/β5 heterodimer while that in the adult brain remains untouched (47). Huang revealed that irisin could promote BDNF expression through an experiment in which diabetic rats were divided into four groups, namely, control, model, irisin (irisin overexpression), and irisin-short hairpin (sh)-RNA (irisin interference). The results confirmed the significantly lower levels of irisin and BDNF in the model and irisin-shRNA groups, and the much higher level in the irisin group. Importantly, irisin and BDNF performed at higher levels in the irisin group compared with the model group, and their expressions were slashed in the irisin-shRNA group compared with the model group (48). The same finding was obtained by Wrann et al., who employed siRNA to interfere with FNDC5/irisin expression in cortical neurons and found a decrease in BDNF expression (49). Meanwhile, one study found that recombinant irisin could promote BDNF formation by stimulating the cAMP→PKA→CREB→BDNF pathway, which was shared by other studies that confirmed the role of irisin in enhancing BDNF expression by increasing cAMP and phosphorylating CREB (pCREB). Therefore, there is much evidence that irisin can induce BDNF production directly in the brain or *via* the cAMP→PKA→CREB→BDNF pathway, which protects the brain (50).

Brain-derived neurotrophic factor, as a neurotrophic factor, significantly promotes synaptogenesis and plasticity, neuronal cell survival, migration, and dendritic branching. Mainly released by microglia and astrocytes, it is abundantly expressed in those areas of the brain associated with cognition, including the hippocampus, cerebral cortex, amygdala, and cerebellum. Animal models observed the exercise-induced BDNF in different brain regions, the most obvious of which is the hippocampus. Notably, decreased expression levels of BDNF were found in different areas of the brain of patients with AD (51). Other studies have reported the association of BDNF with the mesocortical limbic system in the brain and the importance of BDNF in brain circuits. Besides, hippocampal neurogenesis and hippocampal neural circuits are inseparable from BDNF, which matters much for LTP and learning memory functions. Mychael et al., who supported the finding, injected C57BL/6 mice intraperitoneally with lentiviruses containing two shRNAs, which effectively knocked down FNDC5. The results revealed impaired hippocampal long-term potentiation and memory maintenance in a novel object recognition task (50). Moreover, BDNF underlies neuronal stress resistance and synaptic plasticity by facilitating the differentiation and maturation of developing neurons and enhancing synaptic transmission and plasticity in mature neurons, which contributes to memory formation and learning (52). Recent studies have also found the three approaches adopted by BDNF to regulate synapse formation: increasing axonal and dendritic branching, inducing the formation of axonal and dendritic buckles, and stabilizing existing synapses. Notably, BDNF can promote hippocampal cell proliferation, and so can irisin by increasing the release of BDNF, which regulates the STAT3 signaling pathway. Such a process ultimately leads to a reduced risk of AD. One study found that 100°nmol/L irisin promoted hippocampal cell proliferation by 70–80%, and BDNF expression in FNDC5-transduced primary cortical neurons was increased fourfold compared to the control group (49). Several other studies have also found that irisin may induce the STAT3 signaling pathway through the motor-irisin-BDNF axis to proliferate hippocampal cells (17), supporting irisin as a therapeutic target of AD (53). In summary, irisin passes through the blood-brain barrier with the help of peripheral circulation and eventually enters the brain, which then induces BDNF production directly or indirectly, allowing BDNF to exert protective effects on brain function through various pathways.

### Irisin suppresses neuroinflammation in Alzheimer’s disease

Irisin exerts its anti-inflammatory effects by promoting the expression of BDNF, which binds to its receptor TrkB before the activation of AKT and ERK1/2 signaling pathways and reduces the role of IκBα phosphorylation and NF-κb (54). In addition, irisin curtails the release of inflammatory factors through its action on astrocytes (15). Altered roles of BDNF/TrkB cause neurodegenerative changes, while lower BDNF/TrkB levels incur neuroinflammation. Moreover, irisin promotes the release of inflammatory cytokines IL-1β and IL-6 and triggers the JAK2/STAT3 signaling pathway, which upregulates the C/EBPβ/AEP pathway. Such a process finally results in Aβ precursor protein breakage, followed by Aβ and tau protein and cognitive dysfunction (55). In a word, irisin deficiency decreases BDNF/TrkB levels and induces the formation of Aβ and tau protein, thus exacerbating cognitive dysfunction. The role of irisin on astrocytes by decreasing pro-inflammatory factor release also deserves attention.

Astrocytes (also astroglia), the most numerous and prominent cells in the nervous system, contribute much to neuronal metabolism, the repair of injuries to the brain and spinal cord, phagocytosis and isolation of synapses, and the metabolism of neurons passing through astrocytes. Irisin protects cultured neurons by modulating astrocytes and preventing neuronal cells from Aβ. One study found that the treatment of astrocyte-conditioned cultures with irisin for approximately 12 h protects the neurons from the toxic effects of Aβ (56), which is shared by an *in vitro* study. Despite a potential neuroprotective impact of irisin on primary neuronal cultures after Aβ injury, no protection of neuronal cells was observed when cultured neurons were treated directly with irisin or Aβ. The cells, however, were under protection when they were treated with irisin and Aβ protein and co-cultured with astrocytes. Several other studies also demonstrated that irisin significantly increased neuronal cell viability after treatment with astrocyte-conditioned cultures, which achieves its neuroprotective effect by reducing the release of IL-6 and IL-1β from cultured astrocytes and decreasing COX-2 expression and AKT phosphorylation. For example, Wang et al. found that irisin improved memory and cognition in diabetic mice by decreasing IL-6 and IL-1β expression in their hippocampus (56). Given its protection of neurons by reducing the release of pro-inflammatory factors through various pathways, irisin may be a new target for AD prevention or treatment.

### Irisin intervenes in Alzheimer’s disease by improving insulin resistance and glucose homeostasis

Multiple studies have found that irisin can improve insulin resistance and glucose homeostasis through pathways of PI3K/Akt signaling and p38 mitogen-activated protein kinase (p38MAPK). Animal experiments proved the role of overexpressed FNDC5/irisin in mice fed a high-fat diet in improving insulin resistance and lowering blood glucose, a finding supported by Tang, who treated mouse hepatocytes cultured with irisin at high glucose levels and confirmed that irisin could decrease intracellular insulin resistance, increase glycogen synthesis, and increase the number of surviving cells (57). Liu verified the ability of irisin in promoting BDNF expression, followed by the activation of the PI3K/Akt signaling pathway, which controls neuronal glucose homeostasis, mitochondrial biogenesis, and integrity. Higher insulin resistance emerges finally (58). The p38MAPK signaling pathway is also adopted by irisin to improve insulin resistance, and the inhibition or knockdown of p38MAPK inhibits irisin-induced glucose uptake and promotes insulin resistance. Exercise increases reactive oxygen species (ROS) levels, which activates p38MAPK. PGC-1α, regulated by p38MAPK, increases irisin secretion by promoting FNDC5 expression, and irisin acts on white adipocytes and stimulates UCP1 expression, which promotes betatrophin secretion and β-cell regeneration and reduces insulin resistance. Therefore, the ROS→p38MAPK→PGC-1α→irisin→betatrophin→β-cell regeneration pathway may reduce insulin resistance. Ye verified the dependence of irisin-mediated reversal of IR on p38MAPK and that the inhibition of p38MAPK *via* SB203580 significantly reduced irisin-mediated glucose uptake, whereas inhibition of the ERK MAPK pathway *via* U0126 produced a slight blocking effect (59). Therefore, irisin can improve insulin resistance *via* the signaling pathways of PI3K/Akt and p38MAPK and enhance the AD brain’s glucose metabolism and the role of insulin.

## Conclusion

**FIGURE 1:**
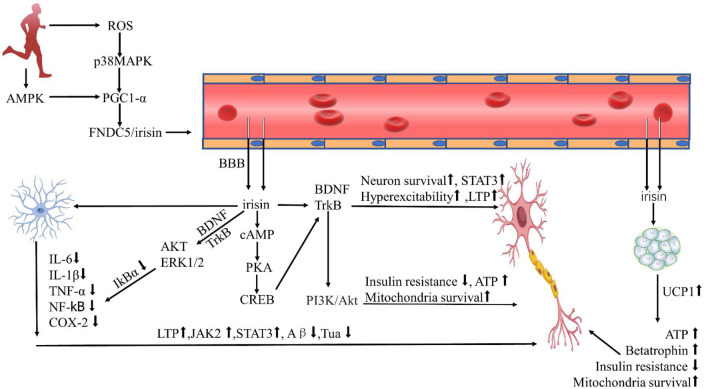
Protective effect of irisin against Alzheimer’s disease. ROS, reactive oxygen species; AMPK, AMP-activated protein kinase; PGC-1α, PPARγ coactivator 1α; FNDC5, fibronectin type III domain-containing protein-5; BDNF, brain-derived neurotrophic factor; cAMP, cyclic adenosine monophosphate; PKA, protein kinase A; CREB, cAMP-response element binding protein; P13K, phosphatidylinositol 3 kinase; Akt, protein kinase B; ERK1/2, extracellular signal-regulated kinase1/2; IL-6, interleukin-6; IL-1β, interleukin-1β; TNF-α, tumor necrosis factor-α; NF-κb, nuclear factor κb; COX-2, cyclooxygenase-2; LTP, long-term potentiation; STAT3, signal transducer and activator of transcription-3; UCP1, uncoupling protein-1; BBB, blood-brain barrier; ATP, adenosine triphosphate.

In this review, we highlighted several remarks concerning the potential beneficial role of irisin in AD. For example, irisin can enhance cognitive AD by promoting BDNF production, mitigate AD by reducing Aβ protein deposition, reduce neuroinflammation by inhibiting pro-inflammatory cytokine expression, and protect against AD by activating the Akt/ERK1/2 signaling pathway to inhibit oxidative stress or ameliorate cardiovascular disease (Figure 1). The mechanism of irisin against AD facilitates the development and testing of drugs for the treatment or prevention of AD, and further physical benefits to patients with AD who can no longer exercise due to health or locomotor conditions. Studies on irisin intervention in AD, however, still face many challenges, one of which is that many current studies are based on experimental studies despite some registered clinical trials aiming to elucidate irisin’s effects on humans (Table 1). Therefore, the exploration of the relationships between irisin and cognition requires extensive clinical studies and long-term follow-up, and the analysis and comparison of animal experiments and clinical studies are necessary, which will deepen the understanding of the differences in the effects of FNDC5/irisin in mice and humans.

**TABLE 1:** Preclinical and clinical studies on irisin and Alzheimer’s disease and Alzheimer’s disease-related dementia.

Stimulus	Species	End point	Result	Year	References
shRNA	Mice	↑FNDC5	↑Hippocampal synaptic plasticity ↑Memory	2019	(50)
Exercise	Mice	↑FNDC5, PGC-1α, BDNF	↑Memory	2012	(40)
Tail vein injection	Mice	↓Dopaminergic neurons from apoptosis and degeneration	↓Parkinson’s disease	2019	(60)
Propofol injection	Mice	↓EGFR	↓Depression	2020	(61)

End point refers to markers directly involved in the irisin pathway.

## Author contributions

KC: writing, original draft preparation, conceptualization, and editing. KW: supervision, reviewing, validation, and editing. TW: supervision, methodology, reviewing, resources, and validation. All authors contributed to the article and approved the submitted version.
